# Oral Asiatic Acid Improves Cognitive Function and Modulates Antioxidant and Mitochondrial Pathways in Female 5xFAD Mice

**DOI:** 10.3390/nu17040729

**Published:** 2025-02-19

**Authors:** Samantha Varada, Stephen R. Chamberlin, Lillie Bui, Mikah S. Brandes, Noah Gladen-Kolarsky, Christopher J. Harris, Wyatt Hack, Cody J. Neff, Barbara H. Brumbach, Amala Soumyanath, Joseph F. Quinn, Nora E. Gray

**Affiliations:** 1 Department of Neurology, Oregon Health and Science University, Portland, OR 97239, USA; varadas@ohsu.edu (S.V.); soumyana@ohsu.edu (A.S.); quinnj@ohsu.edu (J.F.Q.); 2 OHSU-PSU School of Public Health, Oregon Health & Science University, Portland, OR 97239, USA; 3 Department of Neurology and Parkinson’s Disease Research Education and Clinical Care Center (PADRECC), VA Portland Healthcare System, Portland, OR 97239, USA

**Keywords:** antioxidant, mitochondrial function, NRF2, *Centella asiatica*, asiatic acid

## Abstract

**Background/Objectives:** Extracts of the plant *Centella asiatica* can enhance mitochondrial function, promote antioxidant activity and improve cognitive deficits. Asiatic acid (AA) is one of the constituent triterpene compounds present in the plant. In this study, we explore the effects of AA on brain mitochondrial function, antioxidant response and cognition in a beta-amyloid (Aβ)-overexpressing 5xFAD mouse line. **Methods:** Six- to seven-month-old 5xFAD mice were treated with 1% AA for 4 weeks. In the last week of treatment, associative memory was assessed along with mitochondrial bioenergetics and the expression of mitochondrial and antioxidant response genes from isolated cortical synaptosomes. The Aβ plaque burden was also evaluated. **Results:** AA treatment resulted in improvements in associative memory in female 5xFAD mice without altering the Aβ plaque burden. Cortical mitochondrial function and mitochondrial gene expression were increased in the AA-treated female 5xFAD mice, as was the expression of antioxidant genes. More modest effects of AA on cortical mitochondrial function and mitochondrial and antioxidant gene expression were observed in male 5xFAD mice. **Conclusions:** Oral AA treatment improved cognitive and mitochondrial function and activated antioxidant in Aβ-overexpressing mice. These changes occurred independent of alterations in Aβ plaque burden, suggesting that AA could have translational therapeutic relevance in later-stage AD when plaques are well established.

## 1. Introduction

Alzheimer’s disease (AD) is the most common neurodegenerative disease of the elderly, currently estimated to affect 6.7 million people in the United States and predicted to affect more than twice that number by 2050 [1]. The pathophysiology of AD is characterized by extracellular amyloid-β (Aβ) plaque deposits and intracellular neurofibrillary tangles leading to neuronal loss. These changes are associated with the clinical symptoms of memory loss, cognitive decline and inability to perform daily living activities [2,3]. AD is terminal, and the development of novel therapies is hindered in part by an incomplete understanding of the mechanisms that drive disease onset and progression. 

Mitochondrial dysfunction and oxidative stress (OS) are early events in the AD brain that are believed to contribute to the progression of the disease [4]. The magnitude of metabolic activity in the brain, coupled with limited energy reserves, creates an environment with high levels of reactive oxygen species (ROS) that must be balanced by appropriate antioxidant response to avoid increased generation of OS. During aging and in AD, antioxidant response declines while, at the same time, mitochondrial dysfunction increases [5]. In fact, OS and mitochondrial dysfunction are early events that precede the onset of other symptoms of AD [5,6]. The mitochondrial changes include impaired bioenergetics as well as reduction in the expression of mitochondrial enzymes in the electron transport chain (ETC) [7]. Mitochondria are both the main source of and target for ROS. Normal oxidative metabolism generates ~90% of intracellular ROS, the levels of which are tightly regulated by antioxidant enzymes [8]. However, mitochondrial dysfunction results in even greater production of ROS, which, when coupled to the decreased antioxidant response observed in AD, contributes to neuronal degeneration [9]. Therefore, identifying therapies that target antioxidant response and mitochondrial dysfunction could prove an effective strategy for therapeutic intervention.

The endogenous antioxidant response pathway is regulated by the transcription factor, NRF2 (nuclear factor (erythroid-derived 2)-like2; also called NFE2L2), which activates the expression of cytoprotective enzyme genes through binding of the antioxidant response element (ARE) in the promoter. NRF2 expression and its ARE gene targets are downregulated in AD [6]. Induction of NRF2 has been shown to rescue cognitive deficits in AD mouse models [10,11,12]. NRF2 has also been shown to regulate mitochondrial genes, including glucose-6-phosphate dehydrogenase, the enzymes of the pentose phosphate pathway, malic enzyme 1, and isocitrate dehydrogenase 1 [13], further linking antioxidant response and mitochondrial function. 

The medicinal plant *Centella asiatica* (L.) Urban, (Apiaceae) is an herb with an extensive history of use in Ayurvedic and Chinese traditional medicine to boost memory and enhance cognitive function [14]. There have been small clinical studies that similarly observed beneficial effects of extracts of the plant in both healthy and impaired populations with no reported adverse events [15,16]. *Centella asiatica* is also an example of a therapeutic agent that modulates mitochondrial function and the antioxidant response. Our lab demonstrated that a water extract of *Centella asiatica* (CAW) can activate NRF2 induction both in vitro and in vivo, and, in mouse models of aging and AD, it was shown that activation was accompanied by improved cognitive function in CAW-treated animals [11,12,17,18,19,20,21,22].

The complex chemical nature of whole plant extracts makes clinical translation of standardized CAW formulation challenging. *Centella asiatica* contains four triterpene compounds: asiatic acid (AA), madecassic acid, asiaticoside and madecassoside [16]. Each of these compounds has been shown to elicit antioxidant activity and improve mitochondrial function [23,24,25,26,27,28,29,30,31,32,33], and AA in particular has shown promise as neuroprotective agent [34]. In vitro AA has been shown to protect neuroblastoma cells from oxidative stress, mitochondrial dysfunction and cell death induced by a variety of toxic agents including 1-methyl-4-phenyl-pyridine, lipopolysaccharide, rotenone, aluminum and glutamate [35,36,37,38].

There have also been many reports of the antioxidant, anti-inflammatory, mitochondrial and cognitive-enhancing effects of AA in various models of aging neurodegenerative disease [23,26,37,39,40,41,42,43,44,45,46,47,48,49,50,51]. AA improved cognitive function in scopolamine-treated mice [49]; reduced glutamate-induced cognitive deficits and normalized antioxidant response in the cortex and hippocampus in mice [48], while also improving brain mitochondrial function; reduced neuroinflammation; and prevented kainic acid-induced cognitive impairment in rats [50,51]. Based on these findings, we hypothesize that AA may mediate many of the beneficial effects we have observed in mouse models in regard to aging and AD following CAW treatment.

Although the levels of AA are relatively low in CAW, the concentration of its metabolic precursor asiaticoside is quite high [52]. Studies have shown that, in both humans and rodents, orally administered asiaticoside is rapidly and efficiently converted to AA in vivo, and AA is readily absorbed and detectable in the blood stream [53,54,55,56,57]. To our knowledge, AA has not yet been investigated alone in a cognitively impaired patient population; however, it has an excellent safety profile, having been administered to humans in multiple studies without any notable adverse events [56,58,59,60,61]. These factors underscore its potential use as a therapeutic agent in addition to its possible utility as a means of standardizing CAW for clinical use. 

The present study aims to explore the cognitive, antioxidant and mitochondrial effects of AA in the context of Aβ accumulation using the 5xFAD mice.

## 2. Materials and Methods

### 2.1. Mouse Experimental Diets

AA (Sigma Aldrich, St Louis, MO, USA) was incorporated into AIN-93M diet by Dyets Inc. (Bethlehem, PA, USA) at a concentration of 1% AA by weight in the diet. Diets were sterilized by gamma irradiation (5.0–20.0 kGy) at Sterigenics (Oak Brook, IL, USA). AA administered at 1% by weight was selected based on the results of a dose response study in CF1 mice that resulted in the maximal response of endpoints measured without adverse effects (see Supplementary Materials).

### 2.2. Animals 

For this study, 5xFAD colonies were developed from breeding pairs obtained from The Jackson Laboratory. These mice overexpress human amyloid precursor protein (APP) and human presenilin 1 (PS1) with five mutations associated with familial Alzheimer’s Disease (FAD): the Swedish (K670N, M671L), Florida (I716V) and London (V717I) mutations in APP and two in PS1 (M146L and L286V) [62]. The 5xFAD line develops amyloid plaques at a young age (2 months), with cognitive impairment being evident by 5–6 months [62]. Animals were kept in a climate-controlled environment with a 12 h light/dark cycle and provided with water and diet ad libitum until aged to 6–7 months. All procedures were conducted in accordance with the NIH Guidelines for the Care and Use of Laboratory Animals and were approved by the institutional Animal Care and Use Committee of the Portland VA Healthcare System (ACORP protocol #4469 originally approved 3/22/21, renewal approved 4/6/23). At 6–7 months of age, mice were taken off the standard diet and randomly assigned via a random number generator to be either fed AIN-93M (vehicle diet) or AIN-93M containing 1% AA (*n* = 8–12 of each sex per treatment condition). Previous unpublished pilot work in our lab demonstrated that mice readily switch to the diet containing AA; therefore, in this study, adaptive feeding was not employed prior to the beginning of the experiment. The experimental unit is an individual animal; for the number of animals in each condition, see Supplementary Table S1. The number of animals used was based on our previous experience with the 5xFAD mouse line. The variability in the number of animals with data included in each endpoint reflects technical issues with the assays, sample preparation or quantity of samples available, otherwise all data acquired was used for analysis and none was excluded.

**Figure 1:**
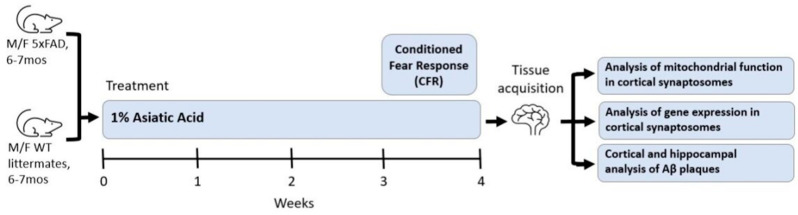
Experimental design; AA treatment in 5xFAD animals.

Treatment continued for a total of four weeks. This duration of treatment, as well as the number of animals, was selected because of our previous work showing that four weeks of treatment with an AA containing extract of *Centella asiatica* was sufficient to elicit cognitive improvements [11,19,21,63], and so this study of the isolated AA compound was designed to match that. In the final week of treatment, mice underwent Conditioned Fear Response testing and then were euthanized via deep anesthetizing with inhaled isoflurane (5% induction with 1–3% maintenance) and cardiac exsanguination; tissue was collected (Figure 1). Plasma was collected in sodium heparin-treated tubes, separated by centrifugation at 3000 rpm for 15 min and then stored at −80 °C until analysis. Researchers were blinded to treatment conditions and genotypes during all analyses, and the order in which animals were analyzed was also randomized to minimize confounders.

### 2.3. Conditioned Fear Response (CFR) Test

The CFR test evaluates contextual memory and has been shown to be affected by inputs from the hippocampus, cortex and amygdala [64]. It has three phases: habituation, conditioning and testing. In the habituation phase, an animal was exposed to a 16 × 16 × 12-inch chamber with a wire floor for 5 min. The conditioning phase occurred immediately following habituation, where the animal was exposed to 3 one-second shocks (0.5 A) randomly distributed over a 3 min period with no more than one shock per minute. The test phase occurred 24 h after the conditioning phase, where the animal was reintroduced once more to the same chamber, but this time not exposed to any shocks. The amount of time spent freezing over a 10 min period is recorded. Freezing time is represented as the change in freezing time from the habituation phase to the test phase to account for any baseline differences in overall activity. Reduced freezing time reflects impaired memory. The CFR is the primary outcome measure of the study.

### 2.4. Synaptosomal Isolation

Cortical tissue was harvested from the mice, and synaptosomes were isolated using SynPer reagent from ThermoFisher (Waltham, MA, USA) according to the manufacturer’s instructions. The total protein content of each synaptosomal preparation was determined by BCA assay. 

### 2.5. Analysis of Mitochondrial Function

Mitochondrial bioenergetics were quantified in isolated cortical synaptosomes taken from the left hemisphere using the Seahorse Xfe96 Analyzer (Santa Clara, CA, USA). A sample of 10 µg of total synaptosomal protein was plated in each well of a polyethyleneimine-coated 96-well plate (*n* = 5–6/animal) and analyzed using the MitoStress kit from Agilent (Santa Clara, CA, USA). The MitoStress kit measures the oxygen consumption rate (OCR) under varying conditions. Three initial baseline measurements were recorded then the ATP synthase inhibitor oligomycin (2 μM) was added and three additional measurements were taken. The difference between the average of these values and the average of the basal respiration reflects respiration is the ATP-linked respiration. Next, p-trifluoromethoxy carbonyl cyanide phenyl hydrazine (FCCP; 2 μM), an ETC accelerator, was added, and 3 more measurements were taken. The average of these values reflects maximal respiration. The difference between maximal and basal respiration is the spare capacity or the extra ability the cell has to deal with unexpected stress. Finally, the ETC inhibitors rotenone and antimycin (0.5 μM) were added.

### 2.6. Gene Expression

RNA was isolated from the remainder of the synaptosomal preparations using Tri-Reagent (Thermo Fisher, Waltham, MA, USA) using the protocol provided by the manufacturer. The Superscript III First Strand Synthesis kit (Thermo Fisher, Waltham, MA, USA) was used to reverse transcribe the RNA and generate cDNA as per the manufacturer’s instructions. Relative gene expression was determined using TaqMan Gene Expression Master Mix (Invitrogen, Waltham, MA, USA) and commercially available TaqMan primers (Invitrogen) for synaptophysin (Mm00436850_m1), post-synaptic density protein 95 (PSD95; Mm00492193_m1), NRF2 (Mm00477784_m1), glutamate-cysteine ligase catalytic subunit (GCLC; Mm00802655_m1), heme oxygenase 1 (HMOX1; Mm00516005_m1), mitochondrially encoded NADH:Ubiquinone Oxidoreductase Core Subunit 1 (Mt-ND1; Hs02596873_s1), mitochondrially Encoded Cytochrome B (Mt-CYB; Hs02596867_s1), Mitochondrially Encoded Cytochrome C Oxidase I (Mt-CO1; Hs02596864_g1), Mitochondrially Encoded ATP Synthase Membrane Subunit 6 (Mt-ATP6; Hs02596862_g1) and glyceraldehyde-3phosphate dehydrogenase (GAPDH; Hs02758991_g1). Quantitative PCR (qPCR) was performed on a StepOne Plus Machine (Applied Biosystems, Waltham, MA, USA) and analyzed using the delta-delta Ct method.

### 2.7. Immunohistochemistry

Immunohistochemistry was performed as previously described [11]. The right hemisphere of each animal was fixed in 4% paraformaldehyde, then passed through a sucrose gradient and frozen. Then, 40-micron frozen coronal sections were cut on a freezing microtome. Sections were incubated with agitation in blocking buffer (100 mM TBS, pH 8.0, 2 mg/mL bovine serum albumin, 2% horse serum, 0.5% Triton X-100) for 2 h, then incubated overnight with a primary antibody directed against Aβ (Invitrogen, beta Amyloid (1–40) Polyclonal Antibody, # 44–136) diluted 1:1000 in blocking buffer. Sections were then incubated for 2 h with biotinylated secondary antibody (1:200, Vector Labs, Burlingame, CA, USA) for 2 h with an avidin-linked peroxidase complex (ABC, Vector Labs), then developed with diaminobenzidine (DAB, Sigma) in PBS. Sections were washed, mounted in Permount (Fisher Scientific, Pittsburg, PA, USA) and cover slipped. Protein expression was quantified in at least three coronal sections from each mouse, representing the anterior, middle and posterior hippocampus and cortex. Hippocampal and cortical areas were traced using a computerized stage and stereo investigator software (Image J version 154d 30, Wayne Rasband, NIH, Bethesda, MD, USA). The Aβ plaque burden was expressed as a percentage of the hippocampus or cortex occupied by these plaques.

### 2.8. Quantification of AA in Plasma Using Liquid–Chromatography Tandem Mass Spectrometry (LC-MS/MS)

Duplicate aliquots (50 µL) of mouse plasma samples were prepared for LC-MS/MS analysis using a protein precipitation method adapted from that of Cheng et al., 2003 [65]. An ascorbic acid solution (1%; 10 µL) was added to limit oxidation during workup. Protein precipitation was achieved by the addition of an acetonitrile–methanol mixture (75:25; 200 µL) containing the internal standard chrysin (0.5 µg/mL). Samples were vortexed and placed at 4 °C for 30 min, then centrifuged (10,000× *g*, 5 min at 4 °C) to sediment proteins and salts. The supernatant was filtered (0.22 µm spinfilter, 10,000× *g* for 5 min at 4 °C) and transferred to HPLC vials for LC-MS/MS analysis. HPLC grade water was added to each vial prior to analysis to achieve a 60:40 ratio of organic solvent to water to optimize peak shape.

LC-MS/MS of AA and internal standard was performed at the Oregon Health & Science University’s (OHSU) Bioanalytical Shared Resource/Pharmacokinetics Core (Portland, OR, USA) using a modification of the method described by Nair et al., 2012 [66]. LC-MS/MS was performed on an Applied Biosystems Q-Trap 4000 LC-MS (Framingham, MA, USA) using a Poroshell 120 EC18 column (3 mm id × 50 mm; 2.7 μm) and a Poroshell ultra-high-performance liquid chromatography (UHPLC) guard column (3 mm id × 5mm, 2.7 µm) (Agilent, Santa Clara, CA, USA). The injection volume was 20 μL and the mobile phase flow rate was 0.42 mL/min. Elution was achieved with a mobile phase of solvent A (water containing 10 mM ammonium acetate and 0.02% ammonium hydroxide; pH 8.5) and solvent B (methanol). The gradient design used began with an initial 2 min increase from 40 to 60% B, followed by 60–95% B from 2 to 3.5 min, held at 95% B from 3.5 to 6 min, returned to 40%B by 6.1 min and re-equilibrated at 40% B from 6.1 to 9 min. AA was detected as its ammonium adduct using positive ion mode electrospray ionization and an MS/MS transition of *m*/*z* 506/453. The internal standard chrysin was detected as the unfragmented molecular ion (*m*/*z* 255/255). Representative chromatograms can be found in the Supplemental Materials.

### 2.9. Statistical Analysis

All analyses were performed using GraphPad Prism 6 and STATA16. Behavioral, bioenergetic and gene and protein expression outcomes were assessed for normality. Those that did not meet assumptions were log transformed. However, even with the transformation, many outcome variables still violated assumptions. Because assumptions required for the Analysis of Variance were not met, we decided to use a nonparametric approach to test for group differences on all variables using the Kruskal–Wallis test. Post hoc pairwise comparisons were assessed using a Dunn’s test of all possible pairs to adjust alpha and account for multiple comparisons. All analyses assess variables in their original unit of measurement. We chose to use Kruskal–Wallis tests even for those variables that did meet assumptions for the parametric approach so that there was consistency across all outcomes for a more balanced interpretation. Because the Kruskal–Wallis test uses medians to test for group differences, we use Box-and-Whisker plots to visually display the data. Brain and plasma AA concentrations were analyzed by two-way ANOVAs and Tukey pairwise post hoc tests.

## 3. Results

### 3.1. AA Treatment Improves Associative Memory in Female 5xFAD Mice

To assess the behavioral effects of AA in the context of Aβ accumulation male and female 5xFAD mice were with 1% AA integrated into their chow for 4 weeks. This concentration was selected based on the results of a dose response study in CF1 mice where 1% elicited the maximal activation of brain mitochondrial function and antioxidant response (see Supplementary Materials). Results from 5xFAD mice treated with 1% AA were compared to animals given a control diet (0% AA) for the same amount of time. Testing of associative memory occurred in the final week of treatment using the Conditioned Fear Response (CFR) test. Tissue was harvested at the conclusion of testing.

**Figure 2:**
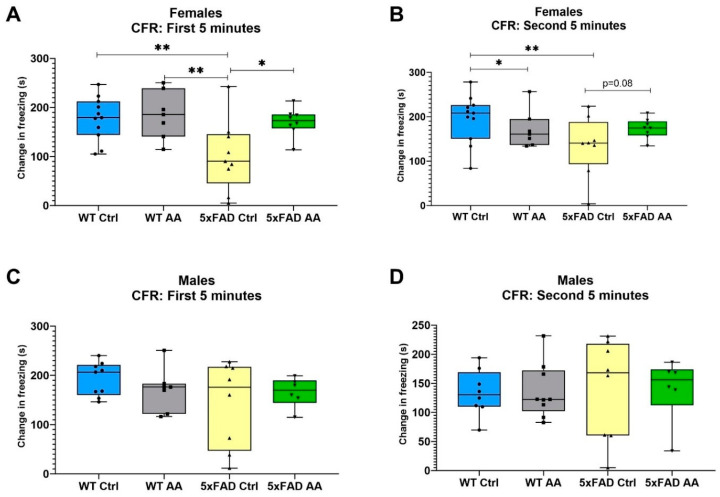
(**A**) A significant improvement in CFR performance is seen in AA-treated 5xFAD female mice over 5xFAD controls in the first 5 min of testing. (**B**) Female 5xFAD AA-treated mice see improvement in CFR score in the second 5 min of testing (*p* = 0.08). Female 5xFAD control mice have a significantly lower change in freezing compared to WT. (**C**) Male 5xFAD and WT AA-treated and control mice do not have significantly different changes in CFR performance in the first 5 min of the test. (**D**) All 5xFAD and WT treatment groups do not have significantly different changes in CFR performance in the second 5 min of the test. N= 6–10 of each sex per treatment condition * *p* < 0.05, ** *p* < 0.01. Symbols within each treatment condition reflect individual data points.

Model significance was tested using Kruskal–Wallis tests followed by a Dunn’s test for post hoc pairwise comparisons. Notably, 5XFAD female mice displayed a significant deficit in CFR performance compared to WT mice in both the first and second five minute stage of the CFR test (χ^2^ = 9.89, *p* = 0.02; χ^2^ = 9.21, *p* = 0.03, respectively). AA treatment significantly improved the CFR performance for the female 5xFAD mice in the first 5 min of the test (Figure 2A), and a similar trend was seen in with AA treatment in the second 5 min for the female 5xFAD mice as well (Figure 2B). No differences in freezing were observed in male mice regardless of genotype or treatment (Figure 2C (χ^2^ = 1.53, *p* = 0.68) and Figure 2D (χ^2^ = 0.46, *p* = 0.93)). 

### 3.2. AA Treatment Does Not Alter Aβ Plaque Burden

**Figure 3:**
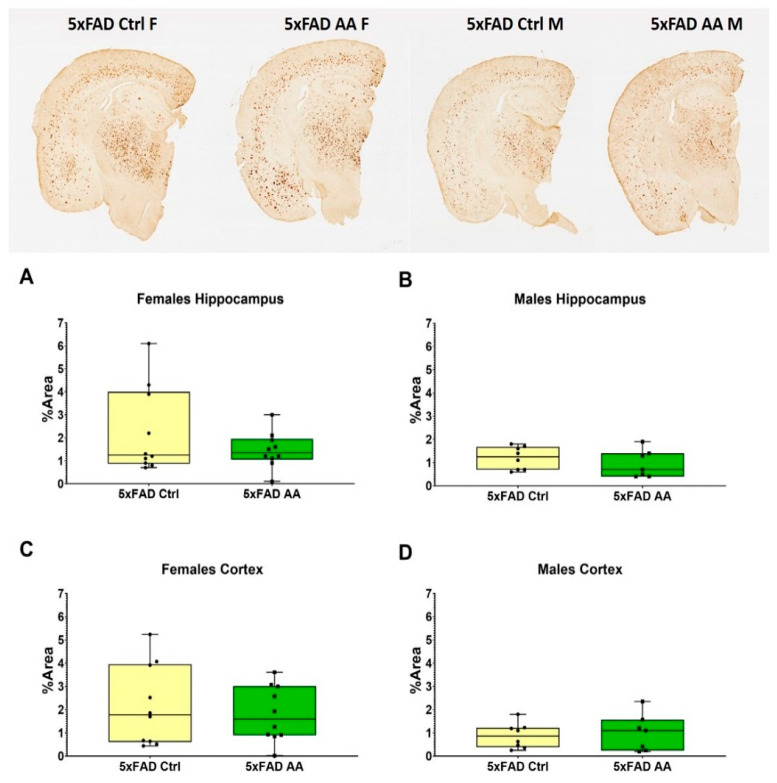
AA treatment does not alter Aβ plaque burden. Aβ plaque levels were not changed by AA treatment in either the hippocampus (**A**,**B**) or cortex (**C**,**D**) of 5xFAD mice. N= 5–7 of each sex per treatment condition. Symbols within each treatment condition reflect individual data points.

Brain tissue from 5xFAD mice was immunostained to quantify Aβ plaque pathology. We found that AA treatment did not alter plaque burden in either the hippocampus (Figure 3A (χ^2^ = 3.65, *p* = 0.16), Figure 3B (χ^2^ = 0.88, *p* = 0.35)) or cortex (Figure 3C (χ^2^ = 2.92, *p* = 0.23), Figure 3D (χ^2^ = 0.10, *p* = 0.75)) of either female or male 5xFAD mice.

### 3.3. AA Treatment Attenuates Deficits in Synaptic Gene Expression in Female 5xFAD Mice

**Figure 4:**
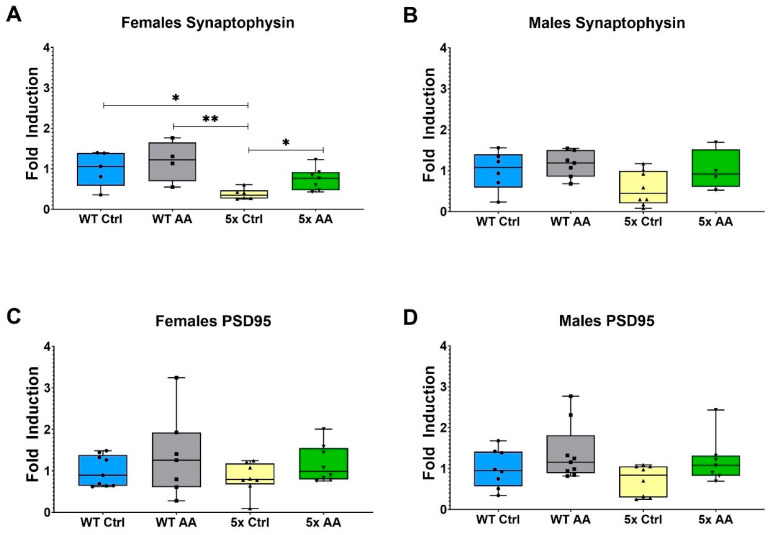
Cortical synaptophysin expression is increased in treated 5xFAD female mice. (**A**) Synaptophysin expression levels in cortical synaptosomes are significantly decreased in female (**A**) and male (**B**) 5xFAD relative to WT controls. AA treatment significantly increased synaptophysin expression in female 5xFAD mice and had a similar but non-significant effect in male 5xFAD mice. (**C**) No significant changes in PSD95 expression were observed in female (**C**) or male (**D**) mice regardless of genotype. N = 4–9 of each sex per treatment condition. * *p* < 0.05, ** *p* < 0.01. Symbols within each treatment condition reflect individual data points.

We observed a reduction in the expression of synaptophysin in cortical synaptosomes isolated from 5xFAD female mice (Figure 4A). AA treatment significantly increased synaptophysin expression in female 5xFAD compared to 5xFAD controls (Figure 4A; χ^2^ = 8.41, *p* = 0.04). Bordering statistical significance, a similar trend was observed with AA-treatment in male 5xFAD mice (Figure 4B; χ^2^ = 7.41, *p* = 0.06). No change in PSD95 expression were observed in either male or female mice regardless of genotype or treatment (Figure 3C (χ^2^ = 2.52, *p* = 0.47) and Figure 4D (χ^2^ = 4.91, *p* = 0.18)).

### 3.4. AA Treatment Improves Mitochondrial Bioenergetics in 5xFAD Mice

**Figure 5:**
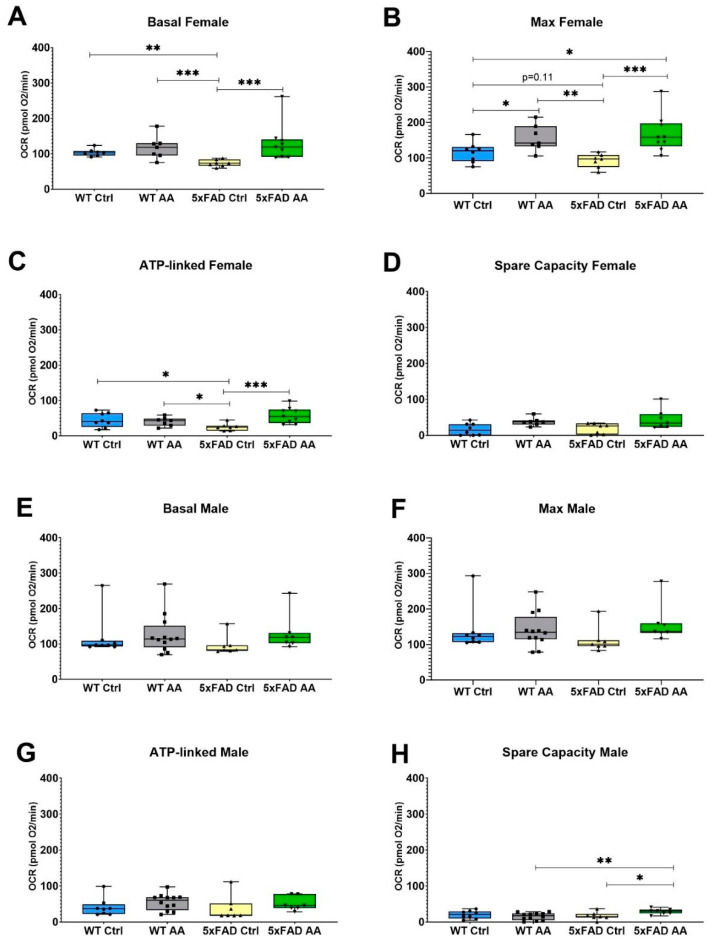
AA treatment improves cortical mitochondrial bioenergetics in 5xFAD mice. AA treatment attenuated deficits in basal (**A**), maximum (**B**) and ATP-linked (**C**) respiration in cortical synaptosomes from female 5xFAD mice. A similar but non-significant trend was also observed for spare capacity (**D**). In male mice, deficits were not observed between 5xFAD and WT mice for any of the bioenergetic metrics (**E**–**H**), although an increase in maximal respiration (**F**) and spare capacity (**H**) was seen in AA-treated 5xFAD male mice. N= 7–12 of each sex per treatment condition. * *p* < 0.05, ** *p* < 0.01, *** *p* < 0.001. Symbols within each treatment condition reflect individual data points.

A significant reduction in basal mitochondrial respiration was observed in cortical synaptosomes isolated from female 5xFAD mice relative to WT mice, which was attenuated by AA treatment (Figure 5A; χ^2^ = 15.48, *p* = 0.002). There was also a significant trend towards diminished maximal respiration evident in female 5xFAD mice that was likewise significantly increased in AA-treated 5xFAD female mice (Figure 5B; χ^2^ = 15.69, *p* = 0.001). ATP-linked respiration and spare capacity were also assessed in cortical synaptosomes isolated from these mice. In female mice, ATP-linked respiration was reduced in 5xFAD mice compared to wild-type mice and was significantly increased with AA administration (Figure 5C; χ^2^ = 10.33, *p* = 0.02). Increased spare capacity in AA-treated WT and 5xFAD female mice approached but did not reach significance (Figure 5D; χ^2^ = 7.57, *p* = 0.06).

The mitochondrial effects of AA were less pronounced in male mice. No significant changes in basal (Figure 5E; χ^2^ = 6.42, *p* = 0.09) or ATP-linked (Figure 5G; χ^2^ = 5.64, *p* = 0.13) respiration were observed in male mice regardless of genotype or treatment. There was, however, a statistically significant increase following AA treatment in spare capacity (Figure 5H; χ^2^ = 8.03, *p* = 0.045) and a borderline significant increase in maximal respiration (Figure 5F; χ^2^ = 7.44, *p* = 0.06) in 5xFAD male mice.

The OCR traces from each treatment group can be found in Supplementary Figures S1 and S2.

### 3.5. AA Increases Expression of ETC Genes in Female 5xFAD Mice

**Figure 6:**
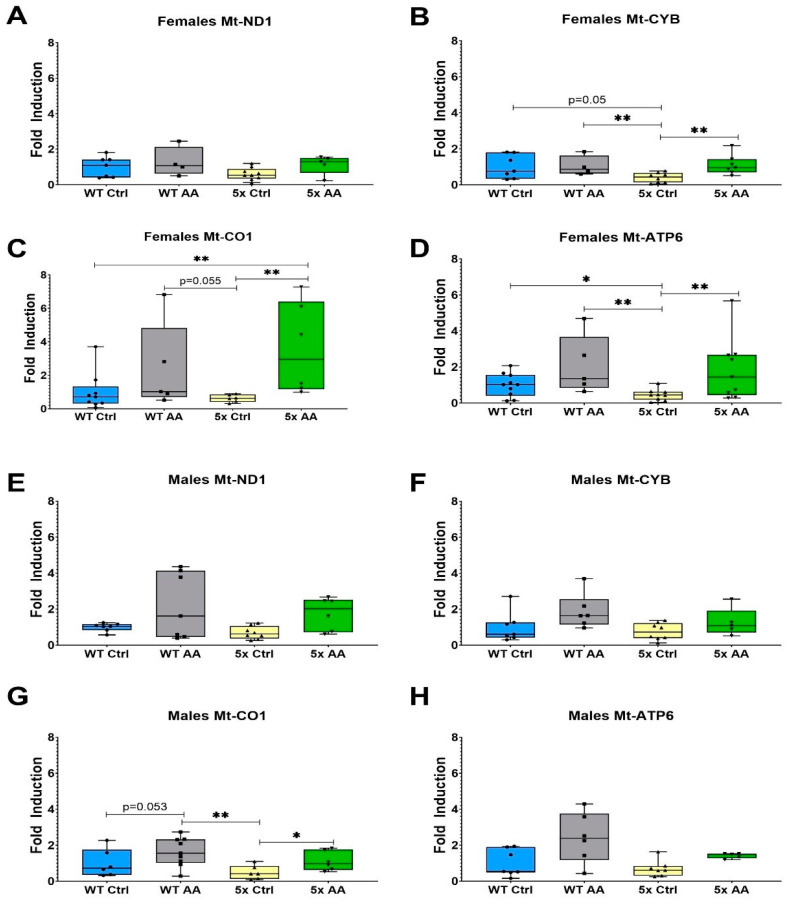
AA treatment induces cortical expression of ETC genes in female 5xFAD mice. ETC gene expression in cortical synaptosomes was quantified in female (**A**–**D**) and male (**E**–**H**) mice. AA treatment increased the expression of Mt-CYB (**B**), Mt-CO1 (**C**) and Mt-ATP6 (**D**) in female 5xFAD mice. In male mice, AA treatment only significantly increased the expression of Mt-CO1 (**G**). N= 4–10 of each sex per treatment condition. * *p* < 0.05, ** *p* < 0.01. Symbols within each treatment condition reflect individual data points.

The expression of the ETC genes Mt-CYB, Mt-CO1 and Mt-ATP6 was reduced in cortical synaptosomes isolated from female 5xFAD mice as compared to WT mice (Figure 6B (χ^2^ = 9.54, *p* = 0.02), Figure 6C (χ² = 8.96, *p* = 0.03) and Figure 6D (χ^2^ = 9.20, *p* = 0.03)). There was no change in the expression of Mt-ND1 in female 5xFAD mice relative to WT (Figure 6A; χ^2^ = 5.13, *p* = 0.16). AA treatment robustly increased the expression of Mt-CYB, Mt-CO1 and Mt-ATP6 in female 5xFAD mice but did not affect the expression of any ETC genes in female WT mice.

In contrast, deficits in ETC gene expression were not observed in male 5xFAD mice relative to WT mice in Mt-ND1 (Figure 6E; χ^2^ = 5.82, *p* = 0.12), Mt-CYB (Figure 6F; χ^2^ = 6.59, *p* = 0.09), or Mt-ATP6 (Figure 6H; χ^2^ = 6.90, *p* = 0.08). However, there was a statistically significant association in Mt-CO1 (Figure 6G; χ^2^ = 8.51, *p* = 0.04). The only ETC gene affected by AA treatment in male 5xFAD mice was Mt-CO1. 

### 3.6. AA Induces Expression of Antioxidant Genes in Female 5xFAD Mice

**Figure 7:**
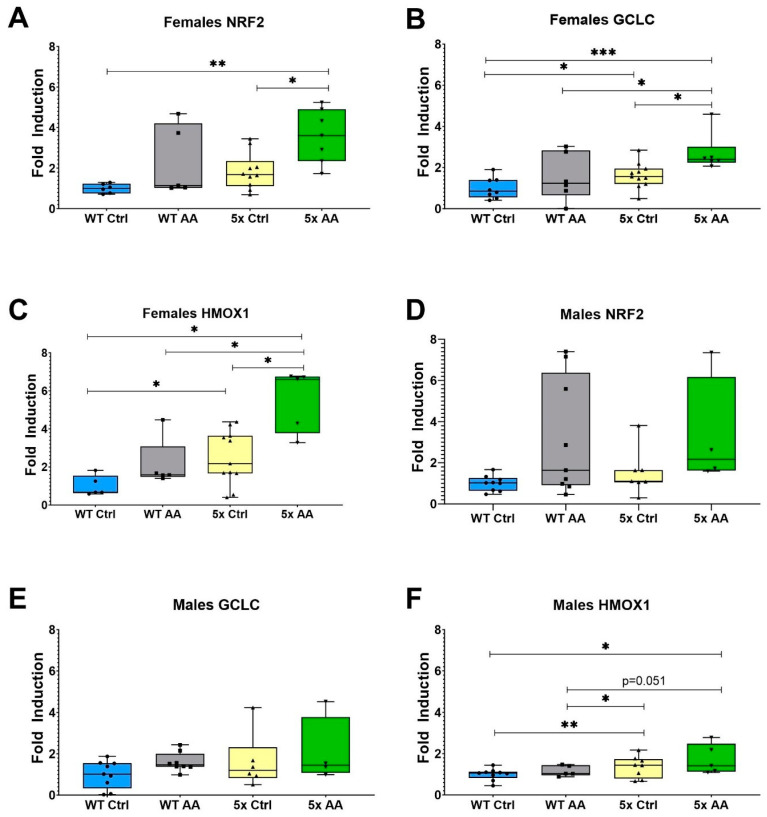
AA treatment induces cortical expression of ARE genes in female 5xFAD mice. AA treatment increased the expression of NRF2 (**A**) and its target genes GCLC (**B**) and HMOX1 (**C**) in female 5xFAD mice. HMOX1 expression was also significantly higher in female WT mice treated with AA than seen in WT controls (**C**). In male mice, there was no significant difference in NRF2 (**D**) and GCLC (**E**) between AA-treated and control mice regardless of genotype. HMOX1 expression was significantly increased in AA-treated WT mice relative to control WT mice (**F**). N= 4–11 of each sex per treatment condition. * *p* < 0.05, ** *p* < 0.01, *** *p* < 0.001. Symbols within each treatment condition reflect individual data points.

The expression of the antioxidant regulatory transcription factor, NRF2, and its target antioxidant genes was increased with AA treatment in cortical synaptosomes from female 5xFAD mice as compared to control female 5xFAD mice (Figure 7A (χ^2^ = 7.84, *p* = 0.05), Figure 7B (χ^2^ = 11.92, *p* = 0.008) and Figure 7C (χ^2^ = 10.02, *p* = 0.02)). There was no effect of genotype or AA treatment on the expression of NRF2 and GCLC in male mice (Figure 7D (χ^2^ = 7.02, *p* = 0.07), Figure 7E (χ^2^ = 2.5, *p* = 0.47). Interestingly, there were differences in the expression of HMOX1 in male mice (Figure 7F; χ^2^ = 8.35, *p* = 0.04), although they were related to genotype and not AA treatment.

### 3.7. AA Concentration in Plasma

The concentration of AA was quantified in the plasma of treated mice (Table 1). In the plasma, there was a significant effect of sex (F (1,33) = 6.547; *p* = 0.015) but not of genotype, nor was there an interaction between sex and genotype (F (1,33) = 1.63; *p* = 0.21). There was substantial variability within each group. 

## 4. Discussion

**Figure 8:**
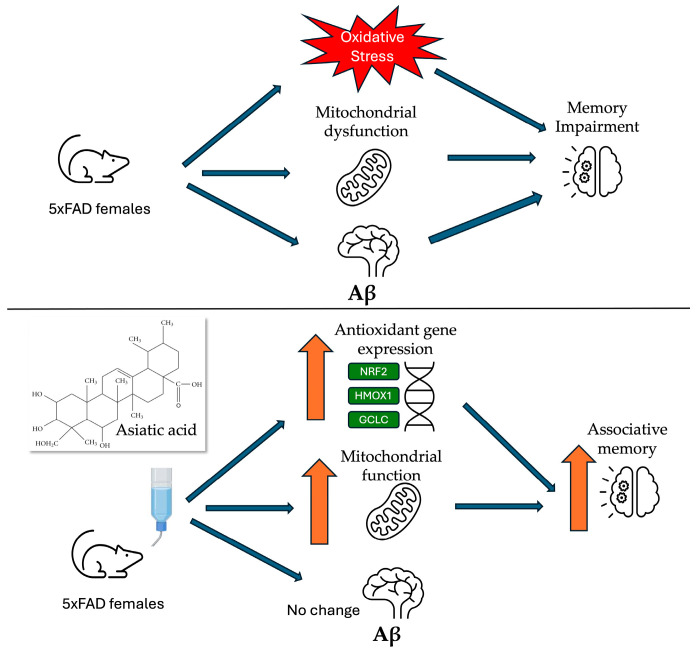
Graphical summary of findings.

*Centella asiatica* has been used in traditional Ayurvedic medicine for over 3000 years and is widely recognized for its cognitive-enhancing applications [67]. Both preclinical and clinical studies have shown therapeutic promise in improving cognitive function [16]. The herb extract is a complex chemical mixture containing several bioactive triterpenes, and the precise compounds that are most responsible for the neuroprotective and cognitive enhancing effects of *Centella asiatica* have yet to be determined. In this study, we have explored the effects of AA, the primary triterpene of *Centella asiatica,* in the 5xFAD model of Aβ accumulation. A high dose of AA (1% in the diet) administered to female 5xFAD mice resulted in increases in brain mitochondrial function and antioxidant response that were accompanied by an improvement in cognitive performance and an increase in synaptic gene expression, without significantly altering Aβ plaque burden (Figure 8). No adverse effects were observed during the course of treatment. This is in line with human studies of AA which have also not reported significant adverse events [56,58,59,60,61].

We found that four weeks of AA treatment significantly increased the cortical gene expression of NRF2-regulated antioxidant enzymes in 5xFAD mice. The NRF2 pathway is an important regulator in the antioxidant response. Oxidative stress is widespread in the AD brain [5], and upregulation of NRF2 has been shown to ameliorate oxidative damage [68]. The effects of AA on NRF2 observed in the present study are in line with previous findings. In vitro, AA treatment of HepG2 cells challenged with tert-butyl hydroperoxide reduced ROS accumulation and apoptosis via NRF2 upregulation [69]. Similar beneficial effects of NRF2 activation by AA have been observed in vivo. Our own lab has previously reported increases in antioxidant response gene expression in the brains of both aged healthy and 5xFAD mice treated with the AA-containing CAW extract [11,12,17,21]. A limitation of the present study is that the protein expression of the antioxidant enzymes was not assessed, and future studies are needed to validate these expression levels via Western blotting or immunohistochemistry. Other groups have reported that AA increases NRF2 protein expression and reduces markers of oxidative stress in rodent models of neurological injury, including doxorubicin-induced toxicities, spinal cord injury and traumatic brain injury [70,71,72]. Future studies confirming the effect of AA on antioxidant enzyme protein expression as well as markers of oxidative damage are warranted. 

A reduction in the number of mitochondria is evident in the AD brain [9]. The fact that, in the present study, AA coordinately increased mitochondrial gene expression suggests that AA could be attenuating this loss of mitochondrial density. This may be due to an effect on mitochondrial biogenesis. While, to our knowledge, this possibility has not been evaluated in vivo, in vitro treatment of a human neuroblastoma cell line with AA resulted in enhanced expression of peroxisome proliferator-activated receptor γ coactivator α (PGC-1α), an important regulator of mitochondrial biogenesis [48]. Future studies evaluating other markers of mitochondrial biogenesis are needed to confirm the effect of AA on mitochondrial content in vivo. 

In this study, we also found the AA treatment improved mitochondrial bioenergetics in the brains of treated mice. These results are similarly consistent with our group’s prior studies evaluating CAW, which found increased expression of ETC genes in the hippocampus of healthy-aged and Aβ-overexpressing mice [11,17]. There are other reports in the literature on the mitoprotective effects of AA specifically as well. AA has been shown to protect mitochondrial membrane potential in rodent liver cells [73,74], and a mouse model of stroke AA treatment resulted in reduced cortical mitochondrial dysfunction [75]. Whether these effects of AA on metabolic function are limited to the brain or are more widespread remains to be seen. In our study, we did not observe any body weight differences between mice treated with AA and the vehicle-treated animals. Food consumption also being similar between groups suggests that AA did not alter global metabolism. However, future studies are needed to confirm the effects of AA on mitochondrial function in different peripheral tissues.

Here, we demonstrated that four weeks of AA treatment improved associative memory in 5xFAD mice. It would be interesting in future studies to assess a broader range of cognitive behaviors and treatment durations. We have reported that 4 weeks of treatment with CAW can improve multiple domains of cognitive function, including learning and executive function, as well as spatial memory [11,19,21]; therefore, we would hypothesize that AA might influence those domains as well. We have also assessed the effects of longer-term CAW treatment and seen that the magnitude of cognitive enhancement is similar to what is achieved after four weeks [12,76]. Additional studies are needed to determine whether this is also the case with prolonged AA treatment. 

Although, to our knowledge, this is the first time AA has been evaluated in a mouse model of Aβ accumulation, the cognitive-enhancing effects of AA that we observed in 5xFAD mice have been well documented in previous studies by our group and others. We have shown that four weeks of CAW treatment increases synaptic gene expression and improves cognitive function in Aβ-overexpressing mice [12,17,21,22]. In a rat epilepsy model of cognitive impairment, pretreatment with AA increased levels of synaptophysin in the hippocampus and improved deficits in learning and memory [50]. Other structurally similar constituent triterpenes from *Centella asiatica* have been reported to similarly attenuate cognitive deficits. Asiaticoside, the metabolic precursor of AA, has been shown to have anxiolytic and anti-depressive effects in rodent models, while madecassoside has been reported to improve memory deficits caused by both lipopolysaccharide and D-galactose [25,29]. Moreover, the cognitive-enhancing effects of other polyphenol compounds, including from coffee, grapes, and blueberries, have likewise been demonstrated in multiple mouse models of cognitive impairment including models of Aβ accumulation as well as in healthy and cognitively impaired human populations [77,78,79,80,81,82]. Interestingly, the effects of AA were consistently more pronounced in female 5xFAD mice than in males across virtually all of the endpoints measured. This variability in response between sexes is in line with our previous studies of CAW in 5xFAD models where we also observed a greater response to CAW in female animals [12,21]. One possible explanation for these sex differences could be related to the greater plaque burden seen in 5xFAD female mice. In our study, we observed that the percent plaque area in the female mice was greater than in male mice. This is consistent with what has been reported in previous studies [83,84]. The greater plaque burden in female 5xFAD mice is associated with more pronounced downstream effects, such as cognitive and mitochondrial impairments, which makes it easier to detect a treatment effect than in male 5xFAD mice, where those deficits are more subtle. Future studies could address the discrepancy in pathology by using older males who have a similar pattern of pathology to their younger female counterparts.

Another potential explanation for the sex differences observed could be related to differential actual exposure of AA. Our results from the plasma do show higher concentrations of AA in female mice. All mice were exposed to the same concentration of AA in the diet; however, the fact that female mice are smaller than male mice suggests that they may in fact receive a higher exposure in terms of mg/kg bodyweight. In this study, mice of both genotypes were group housed and, therefore, individual diet consumption could not be determined. However, the amount of diet consumed per cage was tracked along and did not appear to differ based on treatment conditions (Supplementary Table S1). Another possible confounding variable could be potential hormone–drug interactions in the female mice. This could be investigated in future studies using ovariectomized mice to address this potential variability due to sex hormones. Additional work is needed with more rigorously controlled dosing to fully understand the potential sex differences in bioavailability, compound absorption and possible interactions with AA.

While the precise mechanism remains to be identified, results from this study suggest that the clinical population which is likely to benefit the most from AA are females. Considering that over two-thirds of AD cases in the US are females [1], these results do suggest that continued therapeutic development of AA is warranted even if its clinical application may be limited to only one sex. It is worth noting, however, that prior human studies with AA did not report sex differences in the AA bioavailability or modulation of clinical endpoints [56,58,59,60,61], so it is possible that any sex difference in response may be more pronounced in rodent models.

A significant finding of our study is that AA treatment improved cognitive, mitochondrial and antioxidant endpoints without altering plaque burden in the 5xFAD mice. Again, this finding is consistent with our previous CAW studies in Aβ-overexpressing mice where we did not see a change in Aβ levels following treatment [21,22]. This suggests that the improvements we saw following AA treatment are independent of plaque levels per se and that the intervention is instead targeting the downstream consequences of the plaques. An effect of AA independent of plaque removal would indicate that AA has great promise for clinical utility agents, especially given the limited implementation and high costs of Aβ-targeting therapies [85]. The improvements seen in this study suggest that AA could offer benefits to patients at later stages of the disease where plaque pathology is already well established.

## 5. Conclusions

The results from this study suggest that oral AA administration can enhance mitochondrial function, induce antioxidant response and improve cognitive function in Aβ-overexpressing mice. Future studies are needed to optimize the timing of dosing and confirm similar results in other AD models to fully explore its potential as an AD therapeutic agent. Moreover, since mitochondrial dysfunction and oxidative stress accompany cognitive impairment in other neurodegenerative diseases, the effects of AA in conditions beyond AD also warrant further investigation.

## Figures and Tables

**Table 1:** Plasma concentrations of AA-treated mice. Values for control animals were not detected. N = 6 of each sex per treatment condition.

		Plasma (ng/mL +/− SEM)
Females	WT	704.8 +/− 521.6
	5xFAD	1125.4 +/− 849.1
Males	WT	464.1 +/− 317.2
	5xFAD	406.7 +/− 341.5

## Data Availability

The datasets used and/or analyzed in the current study are available from the corresponding author upon reasonable request.

## Supplementary Materials

The following supporting information can be downloaded at the following: https://www.mdpi.com/article/10.3390/nu17040729/s1, Table S1: Number of animals with data included in each endpoint; Figure S1: OCR trace of Seahorse assay in isolated synaptosomes from females; Figure S2: OCR trace of Seahorse assay in isolated synaptosomes from males; Table S2: Average food consumption per cage per day; Figures S3–S6: Representative chromatograms from AA plasma detection, Supplementary methods, Supplementary results, Supplementary discussion.

## Abbreviations

The following abbreviations are used in this manuscript:

AD	Alzheimer’s disease
Aβ	Beta-amyloid
OS	Oxidative stress
ROS	Reactive oxygen species
ETC	Electron transport chain
NRF2	Nuclear factor (erythroid-derived 2)-like2
ARE	Antioxidant response element
CAW	*Centella asiatica* water extract
AA	Asiatic acid
APP	Amyloid precursor protein
PS1	Human presenilin 1
FAD	Familial Alzheimer’s disease
CFR	Conditioned Fear Response
PSD95	Post-synaptic density protein 95
GCLC	Glutamate-cysteine ligase catalytic subunit
HMOX1	Heme oxygenase 1
Mt-ND1	Mitochondrially encoded NADH:Ubiquinone Oxidoreductase Core Subunit 1
Mt-CYB	Mitochondrially Encoded Cytochrome B
Mt-CO1	Mitochondrially Encoded Cytochrome C Oxidase I
Mt-ATP6	Mitochondrially Encoded ATP Synthase Membrane Subunit 6
GAPDH	Glyceraldehyde-3phosphate dehydrogenase
QPCR	Quantitative PCR
LC-MS/MS	Liquid–chromatography tandem mass spectrometry
