# Supplementary material for: Oral Asiatic Acid Improves Cognitive Function and Modulates Antioxidant and Mitochondrial Pathways in Female 5xFAD Mice
Source: Nutrients. 2025 Feb 19;17(4):729. doi: 10.3390/nu17040729 (PMC11858387; doi:10.3390/nu17040729)
Supplement: Supplementary file 1 [file nutrients-17-00729-s001.zip › nutrients-3469124-supplementary.pdf]

| | WT Female | | WT Male | | 5xFAD Female | | 5xFAD Male | |
|---|---|---|---|---|---|---|---|---|
| | Control | AA | Control | AA | Control | AA | Control | AA |
| CFR | 10 | 7 | 8-9 | 8-9 | 9 | 8 | 8 | 6 |
| Mitochondrial respiration | 7-10 | 7 | 8 | 13 | 7 | 9 | 7 | 7 |
| Gene expression | 5-10 | 5-7 | 6-8 | 5-9 | 7-10 | 5-8 | 6-8 | 5-7 |
| IHC | 0 | 0 | 0 | 0 | 10 | 10 | 8 | 7 |

**Table S1: Number of animals with data included for analysis for each endpoint.**

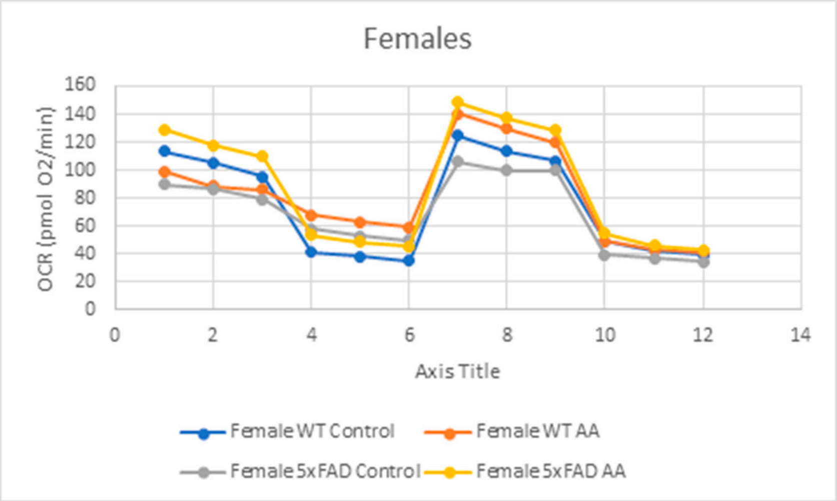


**Figure S1.** OCR trace of Seahorse assay in isolated synaptosomes from females

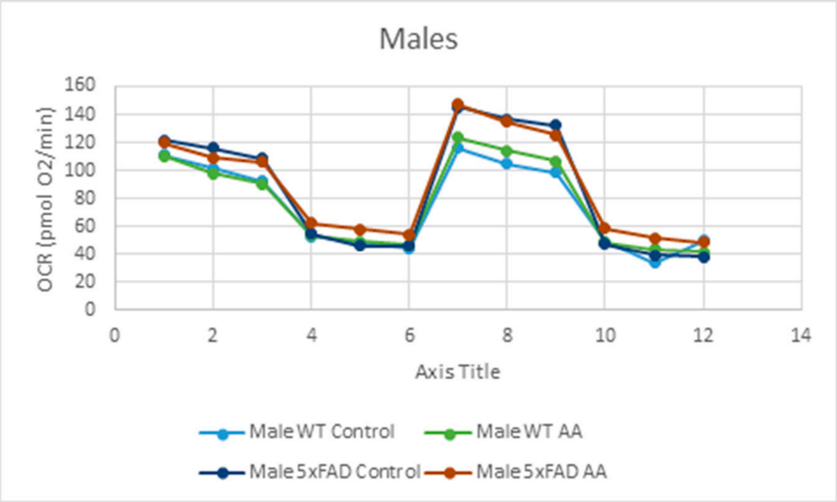


**Figure S2.** OCR trace of Seahorse assay in isolated synaptosomes from males

**Table S2** Average food consumption per cage per day

| | Control | AA |
|---|---|---|
| Females (vehicle) | 3.2g +/- 0.7g | 2.8g +/- 0.8g |
| Males (vehicle) | 3.1g +/- 0.8g | 3.4g +/- 0.6g |

Asiatic acid (AA) in mouse plasma was analyzed using liquid chromatography coupled with electrospray ionization tandem mass spectrometry(LC-MS/MS), with chrysin as internal standard. AA was detected as its ammonium adduct (MS/MS transition m/z 506/453) and chrysin as the unfragmented molecular ion (MS/MS transition m/z 255/255). Sample chromatograms are shown below:

Figure S3: Extracted ion chromatograms of mouse plasma calibration sample A2 containing no asiatic acid (0 ng/ml ) and crashed with internal standard chrysin solution (0.5 μg/mL). Chrysin is the green peak (MS/MS transition m/z 255/255), asiatic acid was not detected (MS/MS transition m/z 506/453).

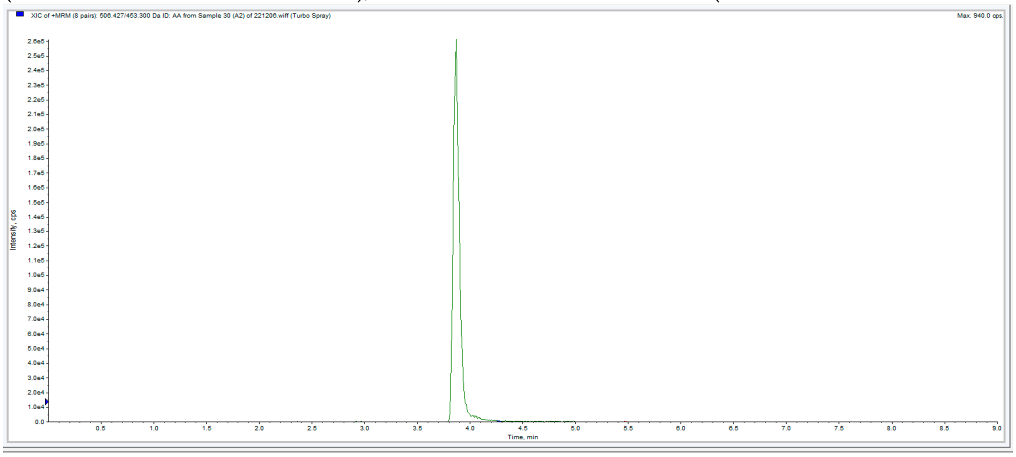


Figure S4: Extracted ion chromatograms of mouse plasma calibration sample E2 containing asiatic acid (600 ng/ml ) and crashed with internal standard chrysin solution (0.5 μg/mL). Chrysin is the green peak (MRM transition m/z 255/255) and asiatic acid is the blue peak (MRM transition 506/453).

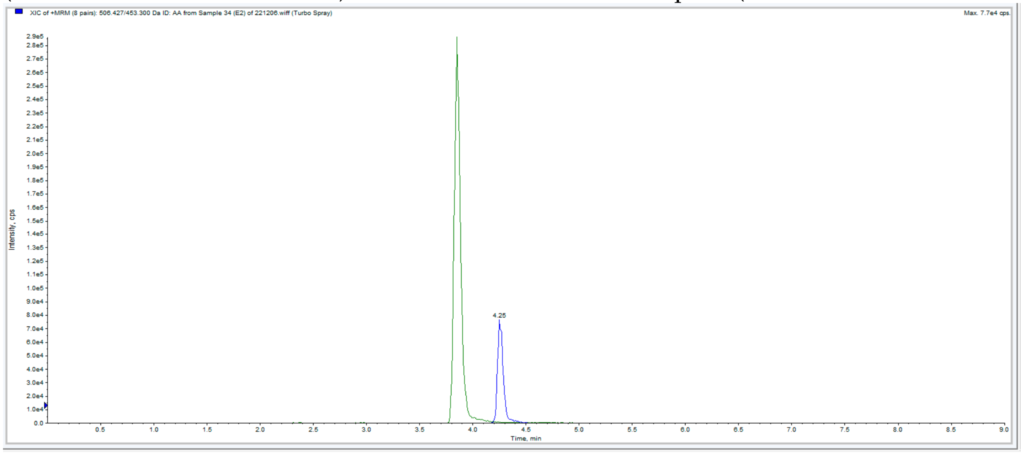

Figure S5: Extracted ion chromatograms of experimental mouse plasma sample 186A from a female 5xFAD mouse treated with control diet (no asiatic acid). Chrysin is the green peak (MS/MS transition m/z 255/255), asiatic acid was not detected (MS/MS transition m/z 506/453).

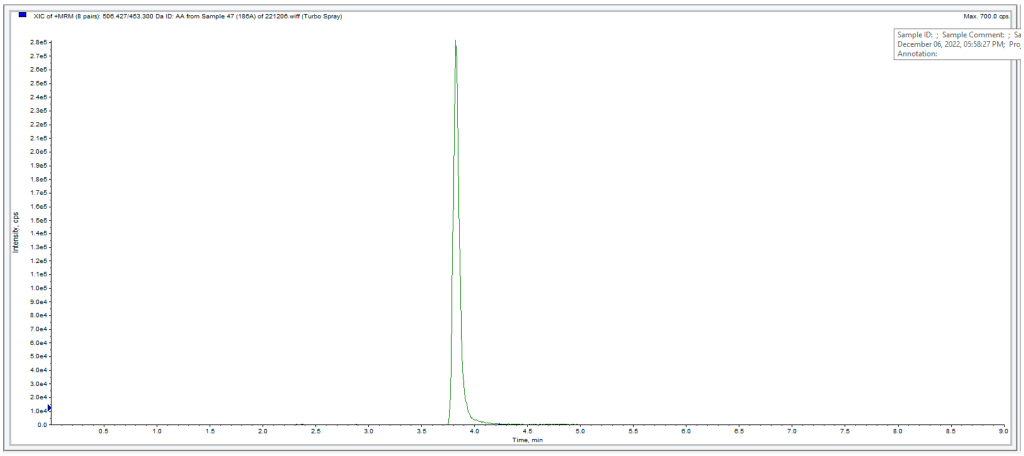


Figure S6: Extracted ion chromatograms of experimental mouse plasma sample 2058A from a female 5xFAD mouse treated with diet containing asiatic acid 1% w/w. Chrysin is the green peak (MRM transition m/z 255/255) and asiatic acid is the blue peak (MRM transition 506/453).

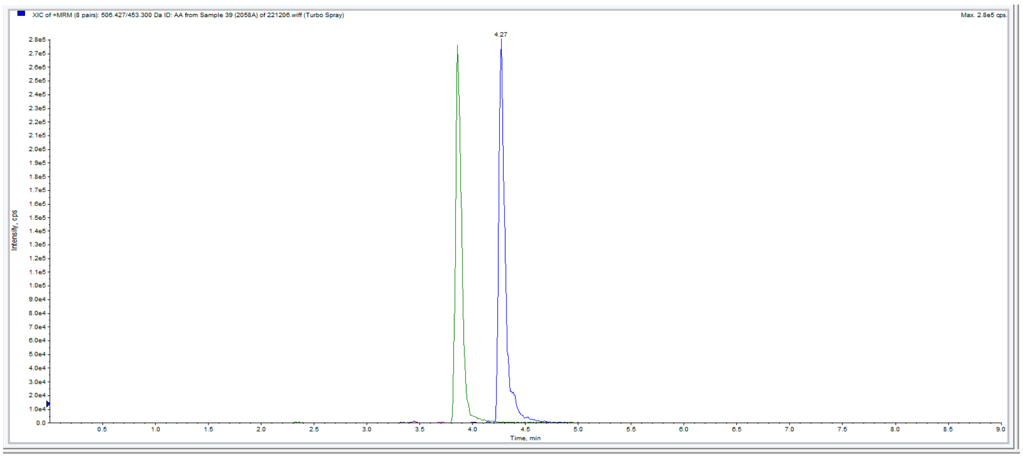


**Supplementary Methods**

*CAW extract*

CAW was prepared as previously described [64]. Briefly, Centella asiatica was obtained from Oregon's Wild Harvest (Redmond, OR) and the water extract was prepared by refluxing 160g of the raw plant material with 2L of water for 2h. This extract was filtered and lyophilized to a powder. A representative sample of both the raw Centella asiatica and the CAW is retained by our lab at -20C. A full description of the chemical composition, determined by targeted liquid chromatography-high resolution tandem mass spectrometry analysis can be found in our previous publication [64].

*Mouse experimental diets*

Either CAW or AA (Sigma Aldrich, St Louis, MO) was incorporated into AIN-93M diet by Dyets Inc. (Bethlehem, PA, USA). CAW was incorporated at 1% w/w to approximate the maximum 1000 mg per kg of body weight per day (mg/kg/d) dose used in our previous dose-response study (21) AA was incorporated into AIN-93M at one of three concentrations: 1) 0.05% AA to match the concentration of AA (w/w) in the 1000 mg/kg/d CAW diet (based on our previously published liquid chromatography-high resolution tandem mass spectrometry analysis of the CAW extract (64)); 2) 0.5% AA to match the concentration of AA+AS (w/w) in the 1000mg/kg/d diet (64) or 3) 1% AA. Diets were sterilized by gamma irradiation (5.0–20.0 kGy) at Sterigenics (Oak Brook, IL, USA).

*Animals*

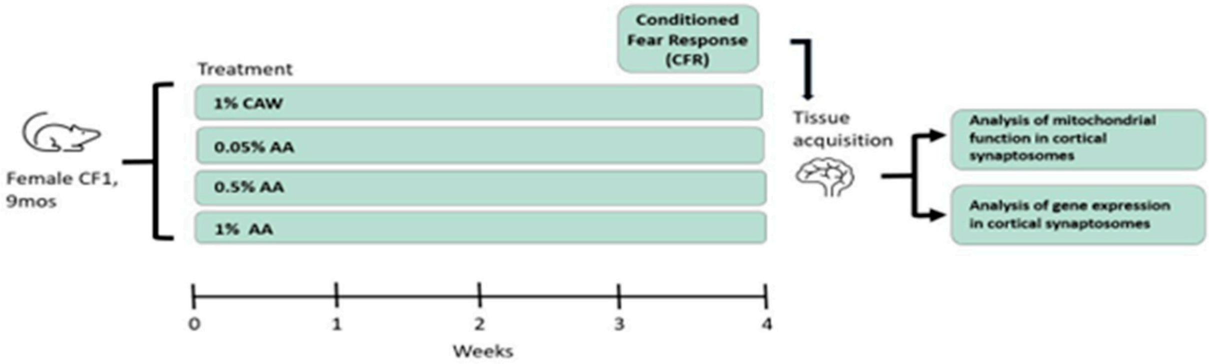


**Figure S7: Experimental design AA dose response in CF1 mice**

Female CF1 mice were obtained from Charles River. Animals were kept in a climate-controlled environment with a 12h light/dark cycle and provided with water and diet ad libitum until aged to 9 months old. All procedures were conducted according to the NIH Guidelines for the Care and Use of Laboratory Animals and approved by the institutional Animal Care and Use Committee of the Portland VA Healthcare System. At 9 months of age mice were taken off the standard diet and fed AIN-93M (vehicle diet), or AIN-93M containing either 1% CAW or 0.05%, 0.5% or 1% AA (n=9-12 per treatment condition). Treatment continued for a total of four weeks. In the final week of treatment mice underwent Conditioned Fear Response testing and then were euthanized and tissue was collected (Supplementary Figure S4).

*Statistics*

All outcome variables were assessed for normality. CFR, distance traveled, basal respiration, ATP-linked respiration and GCLC gene expression were all normally distributed. All other variables did not meet normality assumptions, so a log transformation was performed and normality was established. Time immobile, maximum respiration, spare capacity, NRF2, HMOX1, Mt-ND1, Mt-CYB, Mt-CO1, and Mt-ATP6 analyses are on the log transformation.

We tested the relationship between level of AA (0%, 0.05%, 0.5%, 1%) and all outcome variables. We assessed a dose response by testing for a linear trend using the general linear model (GLM). We created an ordinal variable for AA level as the primary predictor for each outcome variable (see Figures S1-S4). Additionally, we tested for specific group differences between AA levels in addition to a 1% CAW group for each outcome variable. GLM was used to test for model significance followed by post-hoc pairwise comparisons using Tukey and the least square means (lsmeans) statement in SAS. Significance was defined

as $p \leq 0.05$. All bar graphs show means with error bars indicating standard error of the mean with the F and p values listed corresponding to the significance of the linear trend. Analyses were performed using Excel, GraphPad Prism 6, and SAS 9.4.

**Supplementary Results**

*AA treatment dose-dependently increases activity*

To assess the behavioral effects of increasing concentrations of AA, we evaluated overall activity in 9-month-old CF1 mice that were administered AA integrated into their chow at either 0.05%, 0.5% or 1% diet for four weeks. Results were compared to animals given a control diet (0% AA) or a diet 1% CAW for the same amount of time. Testing occurred in the final week of treatment after which tissue was harvested. Distance traveled and time immobile were recorded as metrics of activity during the habituation phase of the CFR (when the animal is freely exploring an open area for 5 minutes).

| | Control (0) | 0.05% AA | 0.5% AA | 1% AA | 1% CAW | F, p |
|---|---|---|---|---|---|---|
| Total distance traveled (m) | 11.55 +/- 2.38 | 15.53 +/- 1.52 | 15.60 +/- 1.85 | 16.36 +/- 1.92 | 15.08 +/- 1.32 | 1.11, 0.37 |
| Time immobile (log s) | 75.15 +/-12.70 | 61.93 +/- 11.02 | 59.39 +/- 14.52 | 38.84 +/- 11.86 | 66.51 +/- 21.26 | 2.27, 0.78 |
| CFR time freezing (s) | 210.6 +/- 31.77 | 222.2 +/- 41.86 | 241.1 +/- 25.43 | 295.5 +/- 20.14 | 289.7 +/- 19.11 | 1.92, 0.12 |
| Synaptophysin (log fold induction) | 1.00 +/- 0.26 | 1.05 +/- 0.19 | 1.01 +/- 0.17 | 1.67 +/- 0.37 | 1.44 +/- 0.22 | 0.34, 0.85 |
| PSD95 (log – fold induction) | 1.00 +/- 0.15 | 0.93 +/- 0.14 | 1.11 +/- 0.18 | 1.66 +/- 0.42 | 1.58 +/- 0.28 | 0.34, 0.85 |
| Basal (pmol O2/min) | 127.57 +/- 3.66 | 131.13 +/- 4.26 | 142.78 +/- 4.56 | 153.31 +/- 8.43 | 143.74 +/- 4.94 | **3.65, 0.01** |
| Maximal (log pmol O2/min) | 198.07 +/- 12.53 | 195.93 +/- 8.92 | 215.09 +/- 8.55 | 235.94 +/- 11.99 | 232.59 +/- 26.22 | **2.72, 0.04** |
| ATP-linked (pmol O2/min) | 36.05 +/- 2.28 | 351.13 +/- 1.88 | 35.77 +/- 2.20 | 42.61 +/- 4.10 | 35.51 +/- 1.92 | 1.42, 0.24 |
| Spare Capacity (log pmol O2/min) | 70.50 +/- 13.17 | 64.80 +/- 5.98 | 72.31 +/- 8.21 | 82.63 +/- 7.68 | 88.84 +/- 16.04 | 0.91, 0.47 |
| Mt-ND1 (log fold induction) | 1.00 +/- 0.23 | 1.16 +/- 0.30 | 1.46 +/- 0.20 | 2.53 +/- 0.69 | 1.65 +/- 0.41 | 1.14, 0.25 |
| Mt-CYB (log fold induction) | 1.00 +/- 0.14 | 1.13 +/- 0.14 | 1.82 +/- 0.31 | 2.22 +/- 0.44 | 1.94 +/- 0.24 | 2.08, 0.10 |
| Mt-CO1 (log fold induction) | 1.00 +/- 0.42 | 1.17 +/- 0.44 | 1.30 +/- 0.38 | 1.85 +/- 0.36 | 1.61 +/- 0.52 | 1.93, 0.13 |
| Mt-ATP6 (log fold induction) | 1.00 +/- 0.24 | 1.16 +/- 0.37 | 1.88 +/- 0.25 | 2.77 +/- 0.73 | 1.96 +/- 0.27 | **4.43, 0.01** |
| NRF2 (fold induction) | 1.00 +/- 0.16 | 1.36 +/- 0.40 | 1.58 +/- 0.49 | 2.41 +/- 0.45 | 1.75 +/- 0.30 | **2.8, 0.04** |
| HMOX1 (fold induction) | 1.00 +/- 0.18 | 1.54 +/- 0.34 | 1.84 +/- 0.32 | 2.35 +/- 0.28 | 1.78 +/- 0.24 | **2.89, 0.05** |
| GCLC (fold induction) | 1.00 +/- 0.15 | 1.40 +/- 0.45 | 1.63 +/- 0.15 | 2.49 +/- 0.47 | 1.95 +/- 0.34 | **2.8, 0.04** |

Table S3. Group differences between AA levels and 1% CAW. Means and standard deviations are reported for all outcomes and groups. The general linear model was used to test for group differences between the control group (0% AA), 0.05% AA, 1% AA and 1% CAW.

The test for differences between groups did not reach statistical significance for either distance traveled or time immobile (Table S3). The dose response test for a linear trend in AA concentration on total distance traveled approached but did not reach statistical significance, (Supplementary Figure S5A). There was, however, a significant linear trend for AA level in relation to time immobile (Supplementary Figure S5B) with time immobile decreasing as AA concentration increased.

*AA treatment increases contextual-associative memory in a dose-dependent manner*

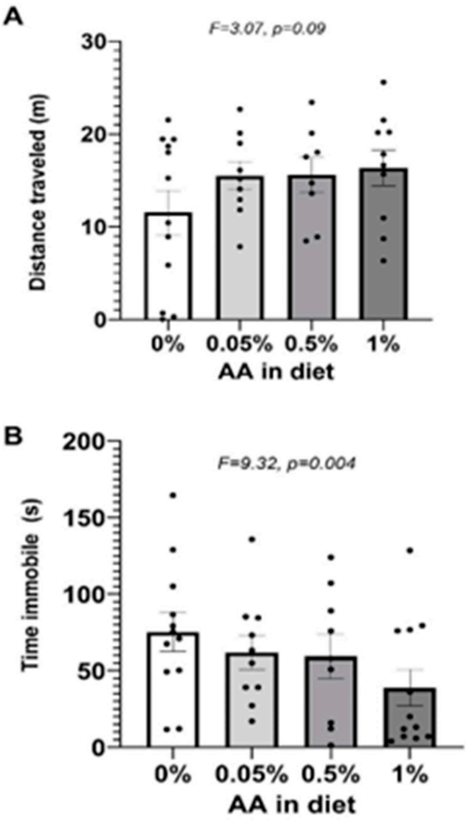


**Figure S8:** . **AA dose dependently increases overall mobility but not distance traveled** A) No significant linear trend was seen with increasing AA concentrations and distance traveled. B) There was a significant linear relationship between time immobile and AA concentration with higher concentrations of AA resulting in a reduction in time immobile.

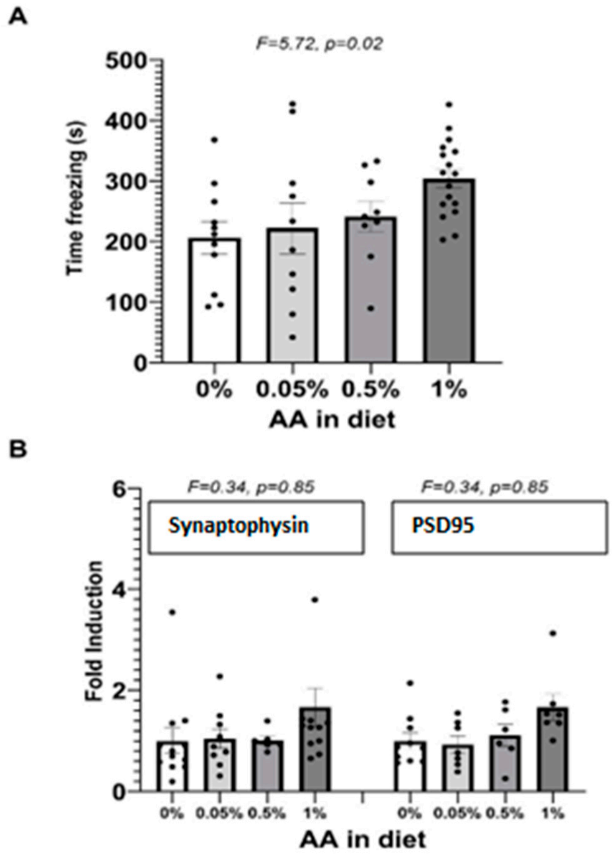


**Figure S9:** . **Associative memory improves in a dose-responsive manner with AA treatment.** A) There was a significant linear relationship between concentrations of AA and CFR response. B) There was no significant linear relationship between expression of synaptophysin or PSD85 and concentration of AA.

Contextual associative memory was evaluated with the CFR test. In the CFR, the animal can freely explore a chamber after which it is exposed to a mild foot shock. The following day the animal is reintroduced to that same chamber and the amount of time that the animal spends frozen is recorded over two five-minute periods. If the mouse remembers the association between the painful stimuli and the chamber, the amount of time frozen will be higher. Because we did not observe any differences in freezing between groups in either 5-minute recording periods the data presented is for the entire 10-minute window. There were no significant group differences between AA treated mice, CAW treated mice and control animals (Table S3); however, we did observe a significant linear relationship between increasing AA concentration and reduced time freezing in CFR performance (Supplementary Figure S6A).

We measured the expression of the synaptic genes synaptophysin and PSD95 in cortical synaptosomes isolated from the brains of treated animals. There were no differences between any of the treatment groups (Table S3) nor was there a significant linear relationship between expression of these genes and concentration of AA (Supplementary Figure S6B).

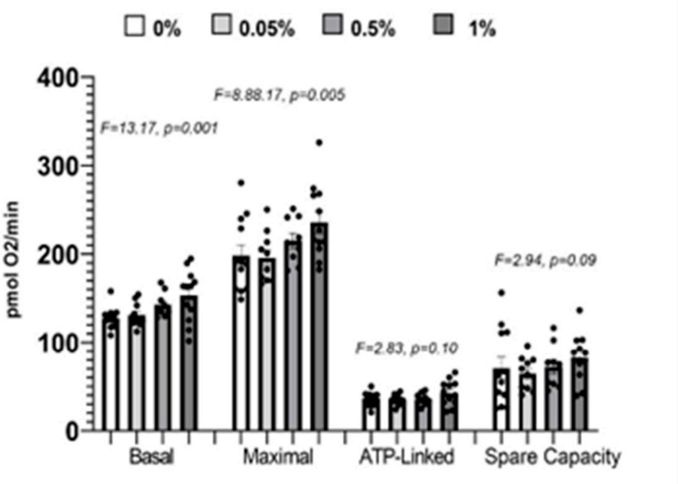


**Figure S10: . AA dose dependently improves basal and maximal mitochondrial respiration in cortical synaptosomes.** Both basal and maximal respiration showed significant linear trends with AA concentration. No significant linear associations were found for ATP-linked respiration or spare capacity

AA alters *cortical mitochondrial bioenergetics*

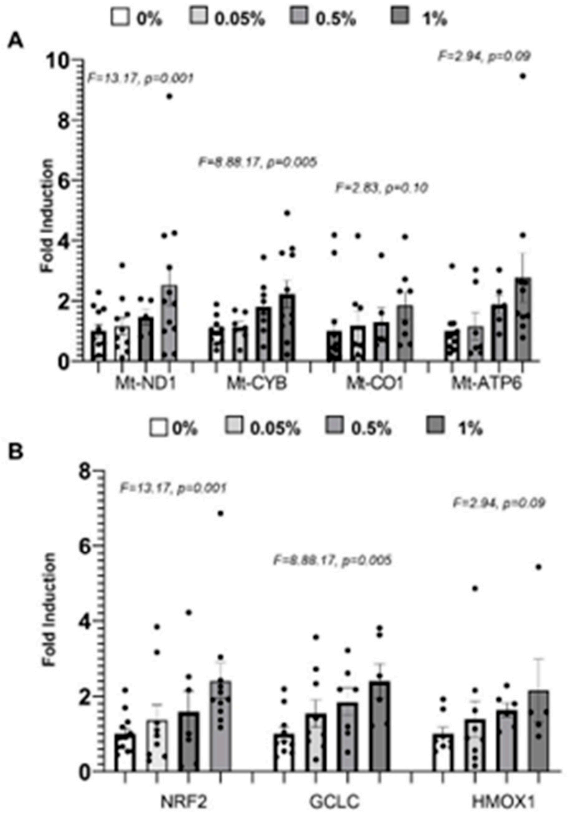


Figure S11: **AA dose dependently increases the expression of mitochondrial and antioxidant genes.** A) All four mitochondrial expressions (Mt-ND1, Mt-CYB, Mt-CO1 and Mt ATP6) in cortical synaptosomes showed statistically significant linear trends with increased AA concentration. B) All three antioxidant gene expressions (NRF2, HMOX1 and GCLC) in cortical synaptosomes showed statistically significant linear trends with increased AA concentration.

The bioenergetics profile of cortical synaptosomes isolated from treated animals was analyzed using the SeahorseXF platform. There was a significant linear relationship between AA concentration and both basal and maximal mitochondrial respiration (Supplementary Figure S7). Additionally, we found significant group differences in these endpoints (Table S3). Post-hoc analyses showed 1% AA significantly increased basal respiration relative to control treated mice. A similar but non-significant increase in maximal respiration between 1% AA treated mice and control mice was observed (p=0.10). CAW treatment did not result in significantly increased basal or maximal respiration.

AA treatment did not affect either ATP-linked respiration or spare capacity. There were no significant linear trends (Figure S7) or significant group differences (Table S3) for ATP production or spare capacity, a metric that reflects the extra energy available for demand increases that occur when cells are subjected to stress.

*AA dose dependently increases the expression of mitochondrial genes in the cortex of treated mice.*

We assessed the expression of the mitochondrial genes Mt-ND1, Mt-CYB, Mt-CO1 and Mt-A TP6 (which encode electron transport chain (ETC) complexes I, III, IV and V respectively) in cortical synaptosomes isolated from the brains of treated animals. All four ETC genes showed a statistically significant linear trend of increased expression with increasing AA concentrations (Figure S8A). However, only Mt-ATP6 yielded statistically significant group differences (Table S3). Post-hoc analyses showed the 1% AA group had significantly higher Mt-ATP6 expression than both the control group and the 0.05% AA group. There was a similar, but non-significant trend towards an increase in Mt-ATP6 expression in the CAW-treated mice as well (p=0.14)

*AA dose increases the expression of antioxidant genes in the cortex of treated mice.*

We also evaluated the expression of antioxidant response genes in cortical synaptosomes isolated from treated animals. We measured expression of the antioxidant regulatory transcription factor NRF2 as well as its antioxidant target genes HMOX1 and GCLC in cortical synaptosomes. We observed a significant linear trend between increased expression of each of the three antioxidant response genes and increasing AA concentration (Supplementary Figure S8B). Although statically significant models were observed for testing group differences (Table S3), the only statistically significant post-hoc pairwise comparison was in GCLC expression showing that mice treated with 1% AA had significantly higher expression than control animals. Again, there was no difference in antioxidant gene expression between the control animals and CAW-treated animals.

**Supplemental Discussion**

It is notable that the effects on mitochondrial function and the expression of NRF2 and its target genes observed in nine-month-old CF1 mice was not seen in the 6-month-old WT littermates of the 5xFAD mice described in the main study. This suggests that the effects of AA may vary depending on the genetic background of the mouse strain and highlights the need for future experiments to assess the intervention across multiple mouse lines.
